# Performance Degradation Behavior and Service Life Prediction of Hydraulic Asphalt Concrete Under Long-Term Water Immersion

**DOI:** 10.3390/ma18153706

**Published:** 2025-08-07

**Authors:** Xinhe Cai, Feng Li, Kangping Li, Zhiyuan Ning, Jing Dong

**Affiliations:** 1 Powerchina Northwest Engineering Corporation Limited, Xi’an 710065, Chinalikangping2017@163.com (K.L.); 2 State Key Laboratory of Water Engineering Ecology and Environment in Arid Area, Xi’an University of Technology, No. 5 Jinhua Road, Xi’an 710048, China; ningzhiyuan1108@163.com (Z.N.); skydongjing@xaut.edu.cn (J.D.)

**Keywords:** hydraulic asphalt concrete, water stability, performance degradation, life prediction

## Abstract

Hydraulic asphalt concrete (HAC) is susceptible to performance deterioration under long-term water immersion. This study conducted compressive, tensile, and bending tests on HAC under various immersion times (0–96 h), established a multidimensional performance evaluation method, and developed a service-life prediction model for long-term water immersion. The average relative error between test values and predicted values was less than 5%, validating the model’s effectiveness and applicability. Results indicate that the rate of mechanical property degradation exhibits stage-dependent characteristics with immersion time, and the water damage resistance of alkaline aggregate is significantly superior to that of acidic aggregate. The predictive model shows that after 192 h of immersion, the retention rate of key mechanical properties for the alkaline aggregate reaches 92.71%, while that for acidic aggregate was only 73.85%. This study establishes a predictive model that provides a theoretical basis for assessing the lifespan of HAC under long-term immersion conditions.

## 1. Introduction

With the advancement of pumped-storage power stations and large-scale water conservancy projects, hydraulic anti-seepage materials are entering a new phase of development. HAC, composed of asphalt, aggregate, and filler, is widely used in impervious facings and core walls of earth–rock dams due to its excellent impermeability [1,2]. However, these core walls and facings are subjected to long-term immersion [3]. Water ingress into the asphalt mixture can impair the bonding between asphalt and aggregates, leading to deterioration in the water stability of HAC. This poses a significant challenge to the safe service of anti-seepage structures [4,5].

In recent years, research on the degradation patterns of water stability in HAC has gained increasing attention. Numerous scholars have conducted in-depth studies from various perspectives. Li et al. [6] focused on modified asphalt concrete, revealing its long-term evolution patterns of water stability and establishing a degradation model. Zhang et al. [7] utilized advanced microscopic observation techniques to thoroughly analyze the microscopic mechanical properties, morphological characteristics, and chemical composition changes in the asphalt–aggregate interface zone, pointing out that the introduction of recycled concrete aggregates weakens the water stability and crack resistance of asphalt mastic gravel mixtures. Sun et al. [8] investigated the impact of replacing traditional limestone powder fillers with Portland cement on the mechanical properties and water stability of asphalt concrete, confirming that good results can be achieved when the replacement ratio is no less than 75%. Ma et al. [9] explored damage accumulation and performance degradation in porous asphalt concrete under varying temperature and humidity, finding that temperature rise aggravates failure development, while moisture damage promotes condensation. Jiang et al. [10] studied the coupled effects of dry–wet cycles and freeze–thaw cycles on the mechanical properties of asphalt concrete under NaCl solution erosion conditions, revealing that the cyclic effects of a saline environment significantly exacerbate the negative impact on mechanical performance degradation. Valentin et al. [11] assessed the effects of various fillers on asphalt–aggregate bond strength, anti-stripping properties, and mixture stiffness, finding that certain industrial waste materials or modified fillers outperform traditional fillers and can improve hydrophilicity. Wang et al. [12] focused on aggregate characteristics, combining photometric colorimetry, water boiling tests, and rheological testing to investigate the mechanisms by which aggregate oxide composition and morphology influence asphalt interface interactions and adhesion, aiming to optimize the evaluation system for aggregate adhesion. In summary, existing research has clearly demonstrated that prolonged immersion in water significantly accelerates the degradation of asphalt concrete’s mechanical properties. Therefore, systematically analyzing the degradation patterns of asphalt concrete’s water stability performance and deeply revealing its underlying mechanisms are the scientific foundation and key to effectively enhancing asphalt concrete’s water stability performance and ensuring the long-term durability of waterproof structures.

Scholars worldwide have conducted extensive and in-depth research on enhancing the water stability of HAC and refining its evaluation methods. Kumlai et al. [13] investigated the effects of incorporating different types of mineral fillers, finding that they enhance the colloidal structure at the asphalt binder scale, thereby improving overall water resistance at the mixture scale. Nobakht et al. [14] established a predictive model for moisture damage at the binder–aggregate interface based on intermolecular bonding forces and adhesion mechanisms at the microscale, providing a theoretical tool for understanding the water damage process. Oldham et al. [15] revealed the mechanism by which the accumulation of acidic compounds at the asphalt–aggregate interface promotes water damage. They proposed using sodium montmorillonite clay additives to passivate the interface and inhibit the nucleation and growth of acidic substances, thereby enhancing water damage resistance. Ren et al. [16] confirmed via CT scanning and water-induced sensitivity testing that incorporating basalt fibers significantly enhances the water damage resistance of asphalt mixtures, thereby improving their structural stability in water-erosion-prone environments. Jiang et al. [17] systematically characterized the water stability of steel slag asphalt concrete through laboratory tests and employed gray correlation analysis to examine the relationship between water stability and factors such as aggregate characteristics, gradation, and asphalt content. Li [18] and Gao [19] focused on the role of anti-stripping agents. Li [18] developed and validated a water damage prediction model for granite-based HAC, while Gao [19] elucidated the key mechanisms by which anti-stripping agents improve asphalt–aggregate interfacial adhesion, enhance water stability characteristics, and increase slope stability. However, despite significant progress in improving water stability, current evaluation systems exhibit notable limitations. Evaluation indicators are predominantly qualitative, lacking core quantitative metrics capable of comprehensively and accurately reflecting the complex water damage process [20].

In summary, to systematically investigate the performance degradation mechanism of HAC under immersion conditions and establish a scientific evaluation system, this study conducted compressive, tensile, and bending tests on HAC specimens subjected to various immersion durations. These tests quantitatively characterized the degradation patterns of mechanical properties as a function of immersion time. Based on gray correlation theory, a quantitative relationship model was established between air void content and key mechanical indicators of water stability. Furthermore, a novel multi-dimensional comprehensive evaluation method was proposed, utilizing the entropy weight method. Finally, by integrating the GM(1,1) gray prediction model with the established performance degradation model, the remaining service life of HAC under long-term immersion conditions was predicted. This approach provides a valuable theoretical basis for ensuring the water stability performance of HAC in engineering practice.

## 2. Materials and Methods

### 2.1. Raw Materials

#### 2.1.1. Asphalt

The study utilized 70# asphalt, with its key performance parameters detailed in Table 1.

#### 2.1.2. Aggregate

As the essential component constituting the skeletal structure of asphalt concrete, the chemical composition of aggregates is a critical factor influencing their adhesion to asphalt. In this section, granite and limestone—commonly used in engineering practice and representative of aggregate types—were selected. The chemical composition of both aggregates was quantitatively analyzed using an X-ray fluorescence (XRF) spectrometer (model: ZSX Primus III+, manufactured by Rigaku Corporation, Tokyo, Japan). The chemical composition data is presented in Table 2.

The alkalinity or acidity of aggregates is classified based on SiO_2_ content, where aggregates with SiO_2_ content below 52% are termed alkaline aggregates, while those exceeding 65% SiO_2_ are classified as acidic aggregates [21]. As shown in Table 2, the SiO_2_ content in limestone aggregates is 1.27%, compared to 76.1% in granite aggregates. Thus, granite constitutes an acidic aggregate, whereas limestone represents an alkaline aggregate.

#### 2.1.3. Mixing Ratio

In the design of coarse and fine aggregate gradation of HAC, the current design specification recommends the Ding Purong gradation formula from Xi’an University of Technology [22,23,24]. The design gradation of HAC is shown in Equation (1):

(1) $$P_{i}=P_{0.075}+(100-P_{0.075})\frac{{\left(d_{i}\right)}^{r}-{\left(0.075\right)}^{r}}{{\left(D_{\max}\right)}^{r}-{\left(0.075\right)}^{r}}$$

where *P_i_* is the sieve opening pass rate, %; *P*_0.075_ is the content of mineral powder with a particle size less than 0.075 mm, %; *d_i_* is the sieve opening diameter, mm; *D*_max_ is the maximum particle size of the aggregate, mm; and *r* is the gradation index.

The gradation index of aggregate is 0.4, the filler content is 12%, the oil-to-stone ratio is 7%, the filler is limestone powder, and 0.8% SCA is added to the acidic aggregate. The particle size distribution of the aggregate is shown in Table 3.

### 2.2. Experimental Methods

#### 2.2.1. Specimen Preparation

Asphalt mixtures were prepared in accordance with the *Test Code for Hydraulic Asphalt Concrete (Beijing, China)* [20]. Compressive specimens were formed using the compaction method. Specifically, cylindrical specimens with dimensions of Φ100 mm × 100 mm were fabricated through single-sided compaction in two layers, with each layer achieving a height of approximately 50 mm after compaction. The number of compaction blows was determined such that the density of the specimens fell within ±1% of the standard Marshall compaction density. After formation, the specimens were stored at room temperature for 24 h prior to demolding. For tensile and bending specimens, the vibration compaction method was adopted. The mixture was poured into molds and compacted uniformly via vibration on a shaking table to ensure dense consolidation. After cooling to room temperature, the specimens were demolded following a 24 h curing period. Subsequently, the specimens were cut into prismatic with dimensions of 40 mm × 40 mm × 220 mm using a double-blade diamond saw, with the cutting speed strictly controlled at ≤8 mm/min. Preparation of all specimens was thus completed.

#### 2.2.2. Specimen Immersion Treatment

The prepared specimens were divided into an experimental group and a control group. Specimens in the experimental group were immersed in a constant-temperature water bath at 60 ± 0.5 °C for 24 h, 48 h, 72 h, and 96 h, respectively, while those in the control group were stored at room temperature.

#### 2.2.3. Measuring Porosity

The porosity of hydraulic asphalt concrete was calculated in strict accordance with the technical requirements specified in *Test Code for Hydraulic Asphalt Concrete (Beijing, China)* [20], and the calculation formula is shown in Equation (2):

(2) $$P=(1-\frac{\rho }{\gamma_{t}})\times 100\%$$

where *ρ* is the measured density of the specimen, (g/cm^3^), and γ_t_ is the maximum theoretical density of asphalt concrete, (g/cm^3^). *ρ* and γ_t_ are calculated according to the specification requirements [20]. Porosity is shown in Table 4.

#### 2.2.4. Measuring Mass Loss Rate

The mass loss rate was determined using the water immersion method [20]. This involved measuring the saturated surface dry mass of the asphalt concrete specimens both before and after immersion in water. The mass loss rate was then calculated using Equation (3).

(3) $$L=\frac{m_{1}-m_{2}}{m_{1}}\times 100\%$$

where *L* is the mass loss rate (%); m_1_ is the surface dry mass of the specimen before water immersion (g); and m_2_ is the surface dry mass of the specimen after water immersion (g).

#### 2.2.5. Test Setup

**Figure 1:**
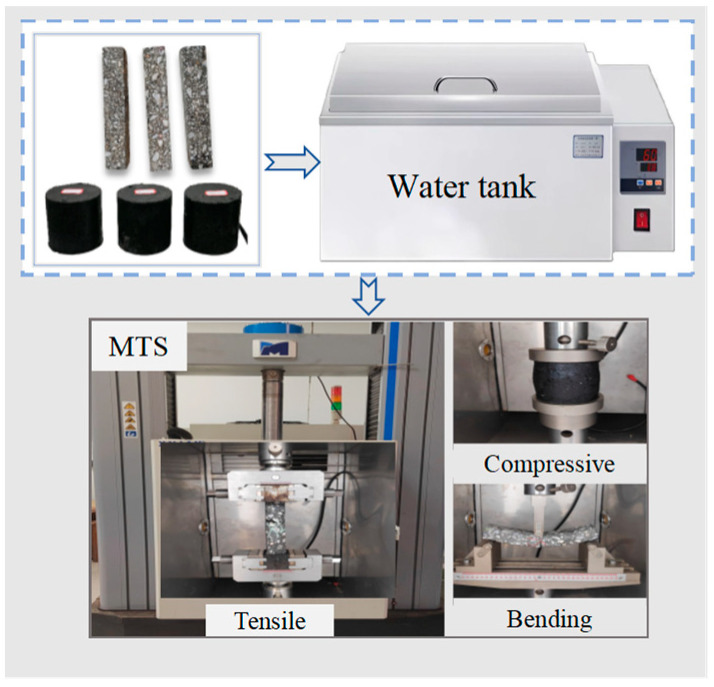
The test flow chart.

The MTS testing machine (Model: MTS809, manufactured by MTS Systems Corporation, Eden Prairie, MN, USA) was employed to conduct compressive, tensile, and bending tests. Tensile tests employed a wedge-type fixture system to ensure reliable axial force transmission and eliminate bending stress. Compressive tests utilized a spherical hinge loading frame with a concave–convex joint structure for load adaptive leveling. Bending tests adopted a three-point bending device with a 160 mm span, with the parallelism error between the loading nose and support rollers controlled within ≤0.1 mm. The test flow chart is shown in Figure 1.

## 3. Analysis of Experimental Results

### 3.1. Compressive Performance

#### 3.1.1. Compressive Mass Loss Rate

**Figure 2:**
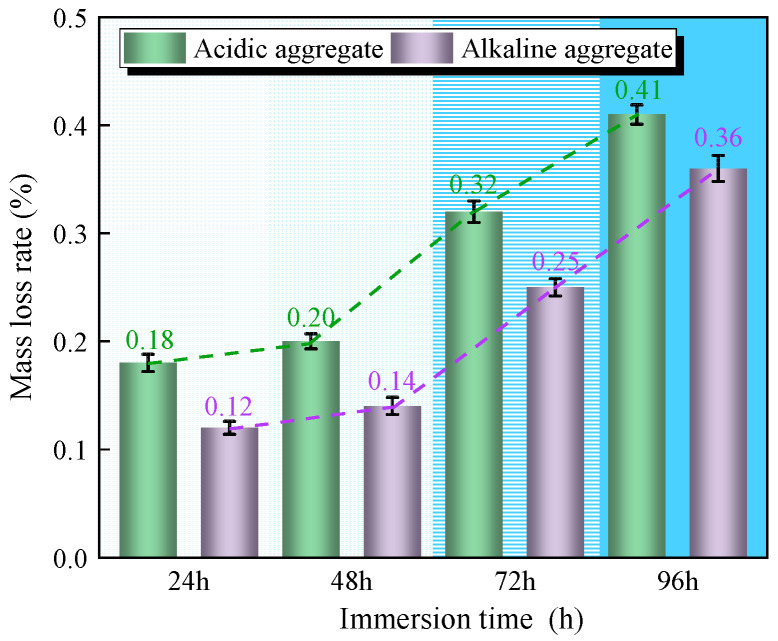
Variation in compressive mass loss rate with immersion time.

The mass loss rates of compressed asphalt concrete specimens under varying immersion duration are presented in Figure 2. As immersion time increased, specimens exhibited progressively higher mass loss rates. After 96 h of immersion, granite specimens showed 0.41% mass loss, while limestone specimens showed 0.36% mass loss. This performance differential is potentially attributable to poorer chemical compatibility between asphalt and granite aggregates, resulting in higher interfacial porosity. When exposed to moisture intrusion, the asphalt film in granite composites experienced greater susceptibility to stripping from aggregate surfaces [25,26].

#### 3.1.2. Compressive Strength

**Figure 3:**
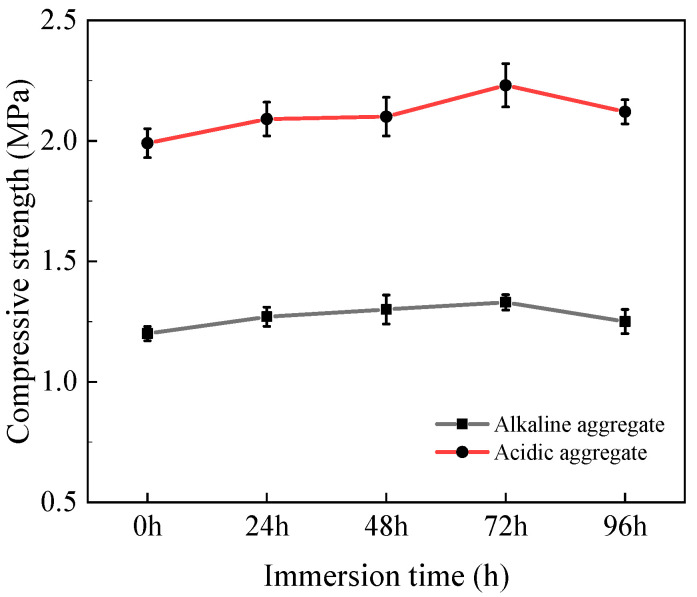
Variation in compressive strength with immersion time.

The compressive strength evolution of asphalt concrete under varying immersion duration is depicted in Figure 3. As immersion time increased, all specimens exhibited progressive strength enhancement. Acidic aggregate asphalt concrete demonstrated a strength growth from 1.99 MPa to 2.12 MPa, increasing by 6.53%, while alkaline aggregate asphalt concrete rose from 1.20 MPa to 1.25 MPa, an increase of 4.17%. This strengthening mechanism likely stems from the temperature susceptibility of asphalt binders. At 60 °C, slight asphalt softening may fill interfacial voids between aggregate and binder, thereby enhancing mortar cohesion [27,28,29]. Furthermore, the consistently higher strength in acidic aggregate asphalt concrete correlates with anti-stripping agents significantly improving interfacial adhesion at acidic aggregate–asphalt interfaces.

#### 3.1.3. Compressive Modulus

**Figure 4:**
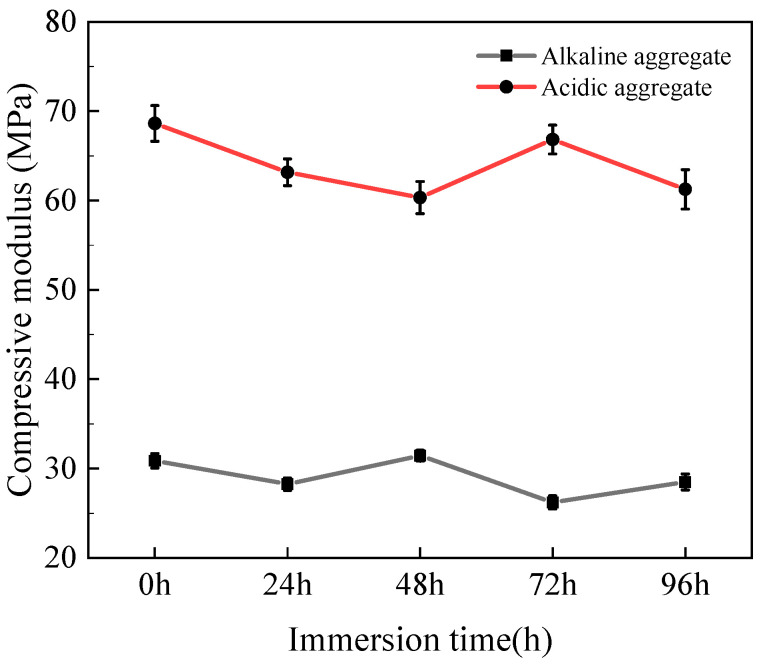
Variation in compressive modulus with immersion time.

The evolution of the compressive modulus in asphalt concrete under varying immersion duration is illustrated in Figure 4. The compressive modulus exhibited progressive reduction with increasing immersion time. Acidic aggregate asphalt concrete decreased from 68.65 MPa to 61.25 MPa (10.78% reduction), while alkaline aggregate asphalt concrete declined from 30.87 MPa to 28.49 MPa (7.71% reduction). This reduction is attributed to structural degradation within the asphalt matrix during prolonged hot-water immersion, resulting in enhanced deformation susceptibility under compressive loading [30,31].

#### 3.1.4. Peak Strain

**Figure 5:**
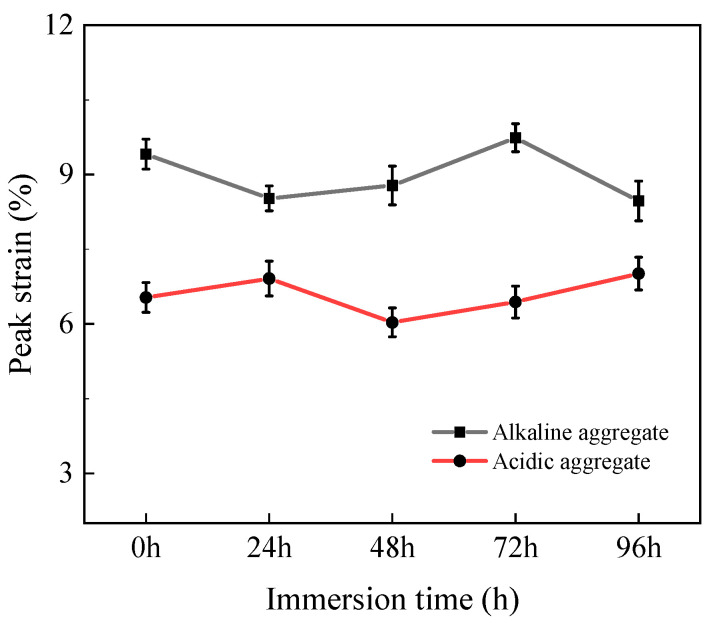
Variation in compressive peak strain with immersion time.

The peak compressive strain evolution of asphalt concrete under varying immersion duration is displayed in Figure 5. With prolonged immersion, acidic aggregate asphalt concrete exhibited an increasing strain trend, while alkaline aggregate asphalt concrete demonstrated decreasing strain behavior. This divergence stems from progressive microstructural deterioration under combined hydrothermal conditions, which typically reduces peak strain. However, in acidic aggregates, silane coupling agent (SCA) incorporation induced molecular spacing expansion within the asphalt matrix, enhancing mortar deformability and consequently increasing peak strain [32].

### 3.2. Tensile Performance

#### 3.2.1. Tensile Mass Loss Rate

**Figure 6:**
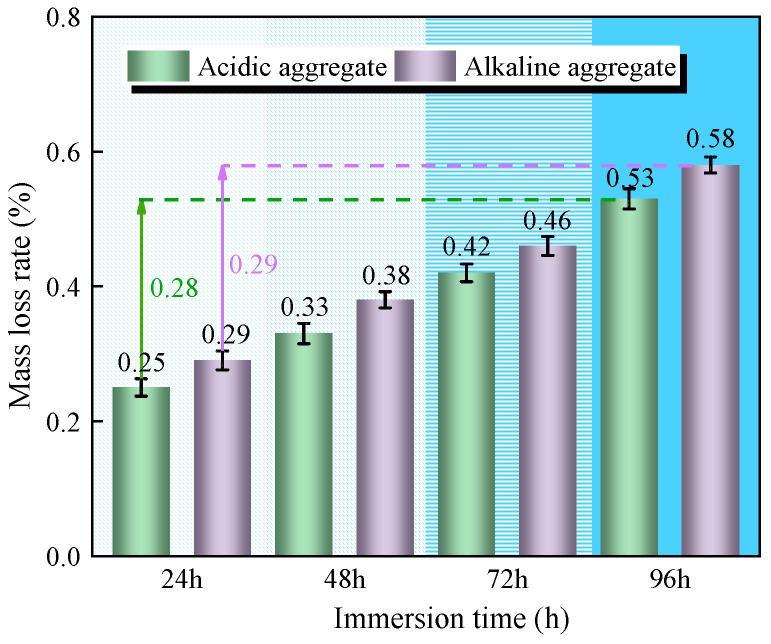
Variation in tensile mass loss rate with immersion time.

Figure 6 presents the mass loss rates of tensile asphalt concrete specimens under varying immersion durations. Progressive mass loss was observed with increasing immersion time. This deterioration mechanism is attributed to moisture intrusion at asphalt–aggregate interfaces, causing asphalt film stripping from aggregate surfaces [25,26,33]. Subsequent aggregate dislodgment reduces specimen mass while degrading asphalt–aggregate adhesion.

#### 3.2.2. Tensile Strength

**Figure 7:**
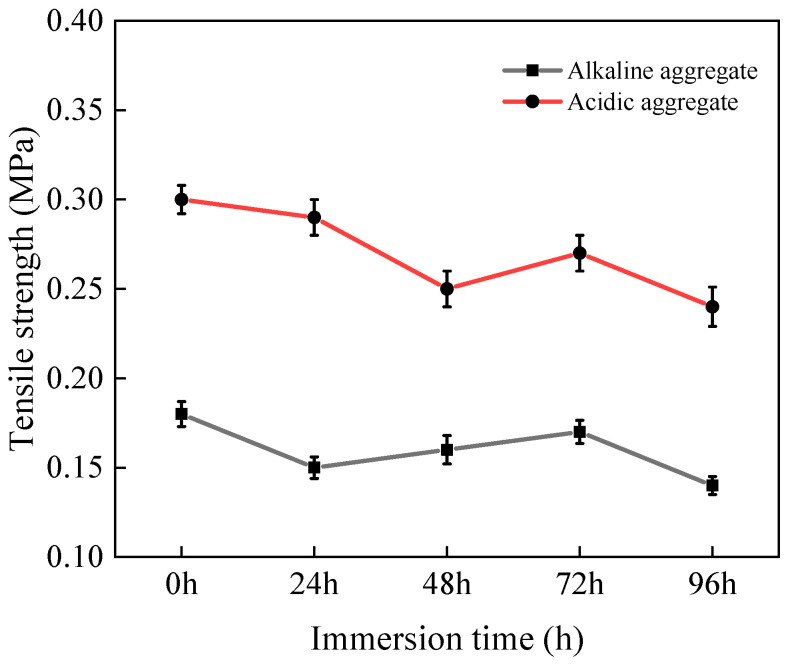
Variation in tensile strength with immersion time.

Figure 7 presents the tensile strength evolution of asphalt concrete specimens under varying immersion duration. Following 96 h of immersion, all specimens exhibited progressive strength reduction. Acidic aggregate asphalt concrete declined from 0.30 MPa to 0.24 MPa (20.0% reduction), while alkaline aggregate asphalt concrete decreased from 0.18 MPa to 0.14 MPa (22.2% reduction). This strength degradation primarily stems from moisture-induced deterioration of asphalt–aggregate adhesion [34,35], which promotes crack initiation and propagation under tensile loading, consequently diminishing resistance to tensile failure.

#### 3.2.3. Tensile Modulus

**Figure 8:**
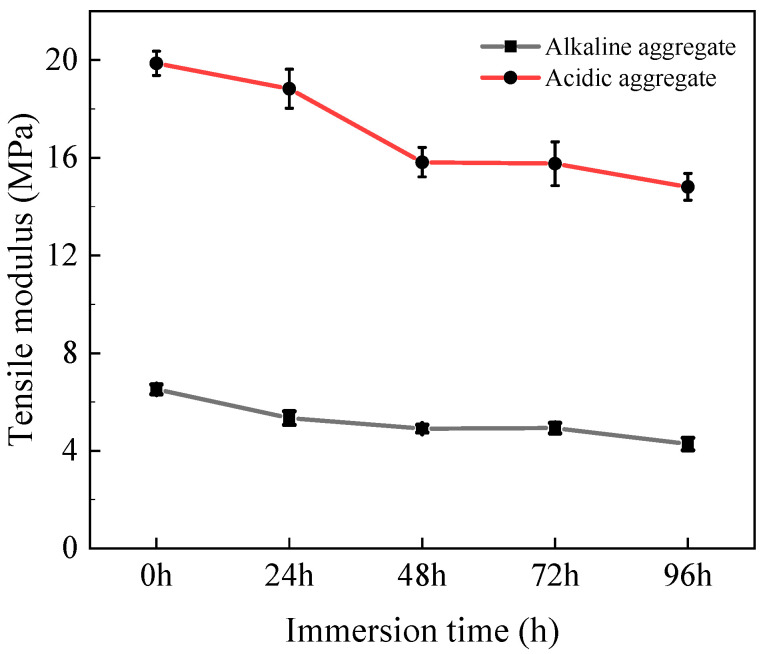
Variation in tensile modulus with immersion time.

Figure 8 illustrates the tensile modulus evolution of asphalt concrete under varying immersion duration and aggregate types. The tensile modulus progressively decreased with extended immersion time. After 96 h of immersion, acidic aggregate asphalt concrete declined from 19.84 MPa to 14.81 MPa (25.35% reduction), while alkaline aggregate asphalt concrete decreased from 6.52 MPa to 4.28 MPa (34.36% reduction). This modulus degradation is primarily attributed to progressive microstructural damage within the material, diminishing its elastic recovery capacity under tensile stress [30,31].

#### 3.2.4. Peak Strain

**Figure 9:**
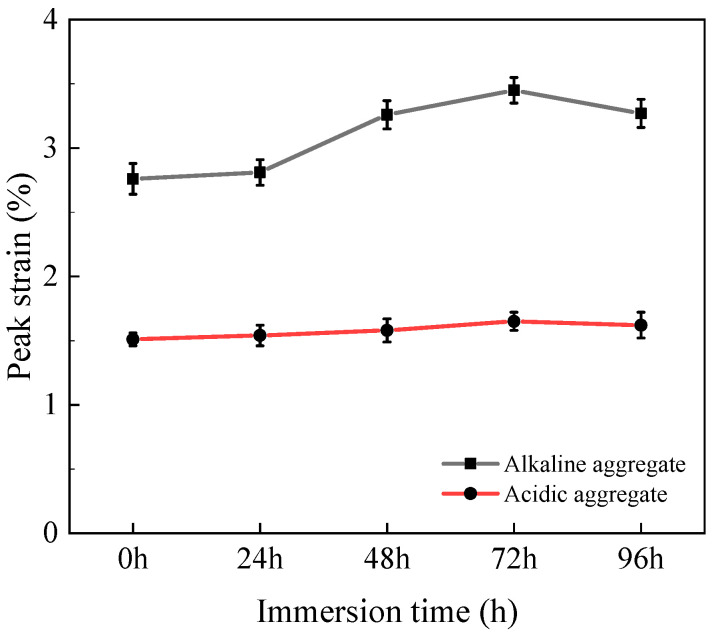
Variation in tensile peak strain with immersion time.

Figure 9 presents the peak tensile strain evolution of high-performance asphalt concrete under varying immersion duration. With prolonged immersion, all specimens exhibited progressive strain amplification. After 96 h, acidic aggregate asphalt concrete increased from 1.37% to 1.67% (21.9% increase), while alkaline aggregate asphalt concrete expanded from 2.53% to 3.57% (41.1% increase). This strain enhancement stems from microstructural deterioration and cohesive strength reduction within the asphalt matrix. The compromised integrity necessitates greater deformation before tensile failure occurs, consequently elevating peak strain values with extended immersion [36].

### 3.3. Bending Performance

#### 3.3.1. Bending Mass Loss Rate

**Figure 10:**
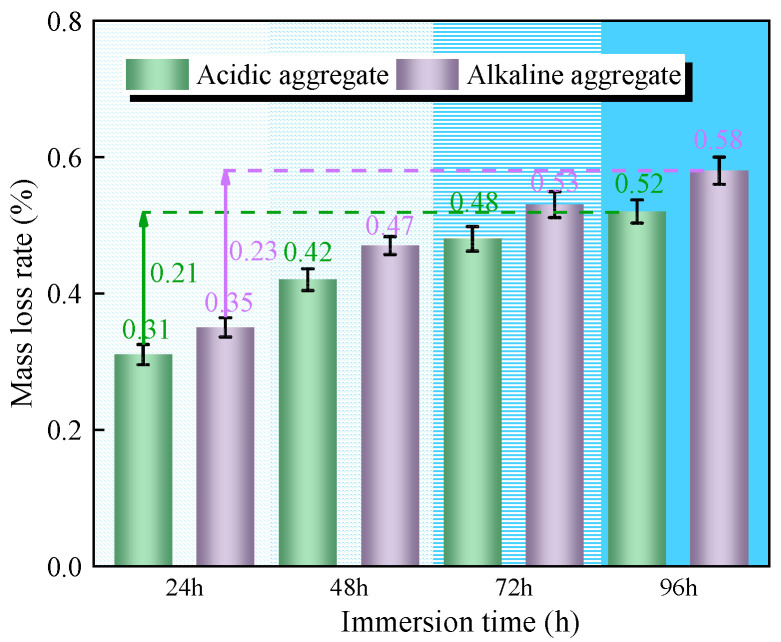
Variation in bending mass loss rate with immersion time.

Figure 10 presents the mass loss evolution of bending asphalt concrete specimens under varying immersion duration. Progressive mass loss was observed with increasing immersion time, attributable to moisture diffusion at asphalt–aggregate interfaces causing surface bitumen stripping. After 96 h of immersion, acidic aggregate asphalt concrete exhibited 0.52% mass loss compared to 0.58% for alkaline aggregate asphalt concrete. The 11.5% lower mass loss in acidic specimens correlates with SCA incorporation, which enhances interfacial adhesion through chemical bonding, thereby reducing moisture-induced deterioration [26].

#### 3.3.2. Bending Strength

**Figure 11:**
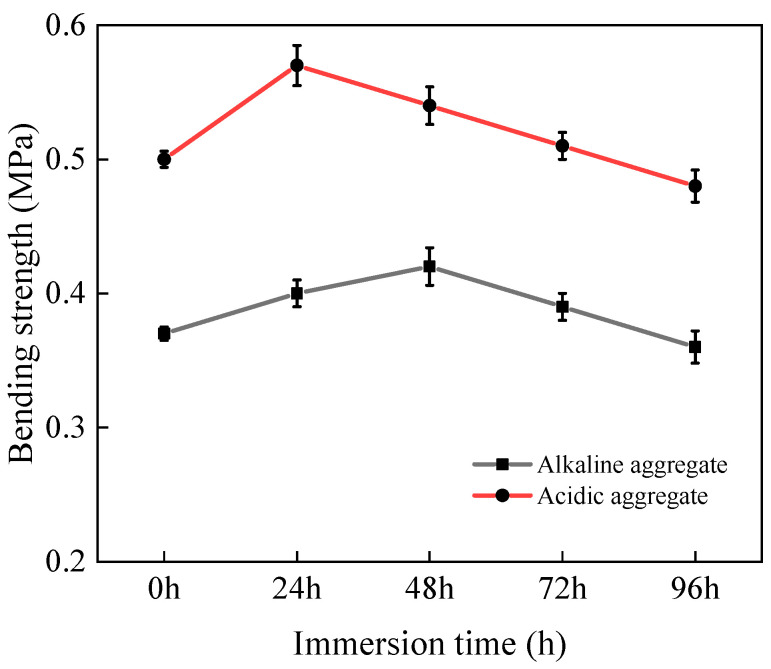
Variation in bending strength with immersion time.

Figure 11 illustrates the bending strength evolution of asphalt concrete under varying immersion duration. Both acidic and alkaline aggregate composites exhibited an initial strength increase followed by progressive degradation after 96 h of immersion. During early immersion stages at 60 °C, thermal conditioning potentially accelerated asphalt aging, inducing binder hardening that temporarily elevated strength [27,37,38]. However, prolonged hot-water exposure eventually compromised material integrity through microstructural deterioration. This degradation reduced resistance to crack initiation and propagation at stress-concentrated zones under bending loading, ultimately diminishing strength performance.

#### 3.3.3. Bending Modulus

**Figure 12:**
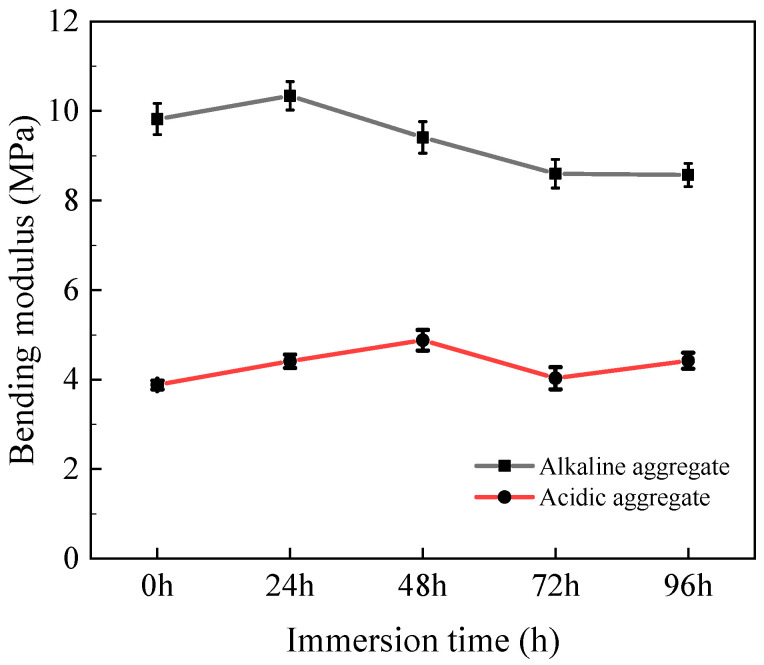
Variation in bending modulus with immersion time.

Figure 12 depicts the bending modulus evolution of asphalt concrete specimens under varying immersion duration. Acidic aggregate asphalt concrete exhibited progressive modulus reduction with extended immersion, attributable to microstructural deterioration that diminished material stiffness. Conversely, alkaline aggregate asphalt concrete demonstrated modulus enhancement due to hydrothermal oxidation of light fractions within the binder [30,31]. This reaction induced temporary binder hardening and increased cementation stiffness, thereby elevating bending modulus.

#### 3.3.4. Peak Strain

**Figure 13:**
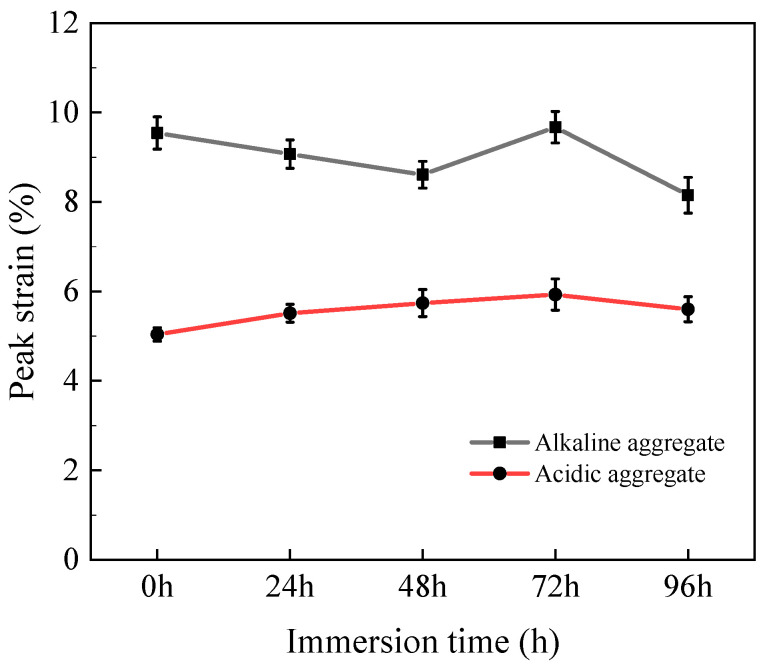
Variation in bending peak strain with immersion time.

Figure 13 presents the peak bending strain evolution of asphalt concrete under varying immersion duration. Acidic aggregate asphalt concrete exhibited increasing peak strain with prolonged immersion, resulting from deterioration of interfacial adhesion. This degradation necessitates greater deformation to reach failure under bending stress, consequently amplifying peak strain values. Conversely, alkaline aggregate asphalt concrete demonstrated decreasing strain trends. This reduction stems from capillary-driven moisture penetration at asphalt–aggregate interfaces, which disrupts physical adsorption between silicate hydroxyl groups on aggregate surfaces and asphaltene molecules, ultimately diminishing interfacial bond strength [39,40].

## 4. Performance Evaluation and Predictive Model

### 4.1. Gray Relational Analysis

In order to investigate the correlation degree between porosity of properties of asphalt concrete, the Gray Relational Analysis is carried out [41,42,43]. Porosity as a function of immersion time is used as a reference sequence, while mass loss rate, peak stress, modulus, and peak strain as functions of immersion time are used as comparison sequences.

To reduce differences in data absolute values, the data sequences are normalized.

(4) $$x_{0}^{\prime }\left(k\right)=\frac{x_{0}\left(k\right)}{x_{0}\left(1\right)}\;\;\;x_{i}^{\prime }\left(k\right)=\frac{x_{i}\left(k\right)}{x_{i}\left(1\right)}$$

where *x*_0_(*k*) and *x*_i_(*k*) are the original data sequences; $x_{0}^{\prime }(k)$ and $x_{i}^{\prime }(k)$ are the sequences after initialization processing; and *x*_0_(1) and *x*_i_(1) are the initial value sequences.

The difference between the initial sequence $x_{0}^{\prime }(k)$ and the comparison sequence $x_{i}^{\prime }(k)$ is calculated to obtain a difference sequence matrix, as shown in Equation (5).

(5) $$\xi_{i}\left(k\right)=\frac{\underset{i}{\min}\underset{k}{\min}\left|x_{0}^{\prime }\left(k\right)-x_{i}^{\prime }\left(k\right)\right|+\rho \underset{i}{\max}\underset{k}{\max}\left|x_{0}^{\prime }\left(k\right)-x_{i}^{\prime }\left(k\right)\right|}{\left|x_{0}^{\prime }\left(k\right)-x_{i}^{\prime }\left(k\right)\right|+\rho \underset{i}{\max}\underset{k}{\max}\left|x_{0}^{\prime }\left(k\right)-x_{i}^{\prime }\left(k\right)\right|}$$

where $\xi_{i}\left(k\right)$ is the correlation coefficient between the i-th comparison sequence and the reference sequence at point *k*; *ρ* is the resolution coefficient, which generally takes values between 0 and 1, and is typically set to 0.5.

The correlation coefficient reflects the degree of correlation between porosity and macro indicators. The larger the correlation coefficient, the closer the correlation between the two. The correlation coefficient formula is shown in Equation (6).

(6) $$\gamma_{i}=\frac{1}{n}\sum_{k=1}^{n}\xi_{i}\left(k\right)$$

where *γ_i_* is the correlation between the reference sequence and the *i*-th comparison sequence; *n* is the total number of data points in the sequence.

**Figure 14:**
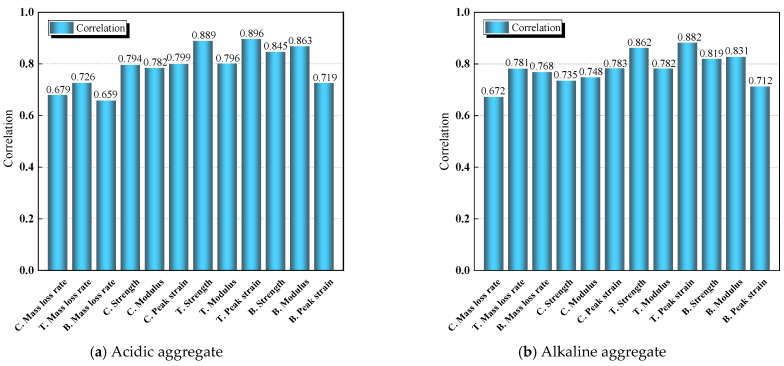
Gray correlation analysis diagram of HAC.

The calculation of correlation coefficients was performed via programming on the MATLAB R2022b platform, and the results have been reflected in Figure 14.

Figure 14 presents the Gray Relational Analysis results comparing the asphalt concrete performance between the two aggregate types. According to gray system theory, relational grades exceeding 0.8 indicate statistically significant correlations. The analysis reveals strong correlations between porosity evolution and key mechanical parameters of water-immersed HAC specimens: tensile strength, bending strength, peak strain, and bending modulus.

### 4.2. Performance Assessment

Asphalt concrete exhibits significant strain variations due to its temperature-sensitive nature under changing test temperatures. Therefore, tensile strength, bending strength, and bending modulus were selected as comprehensive evaluation indicators. The entropy weight method was used to calculate the weights of the three indicators for the water stability of asphalt concrete [44], and a multi-dimensional evaluation index value for water stability was proposed.

The initial water stability evaluation index normalization matrix **A** is constructed as shown in Equation (7).

(7) $$A=\left[\begin{array}{cccc} a_{11} & a_{12} & \ldots & a_{1n}\\ a_{21} & a_{22} & \ldots & a_{2n}\\ \vdots & \vdots & \ddots & \vdots \\ a_{m1} & a_{m2} & \ldots & a_{mn} \end{array}\right]$$

where *m* represents the number of samples, and *n* is the number of water stability evaluation indicators.

The matrix was standardized to ensure that all standardized data were positive numbers. The standardized matrix is denoted as $Z={\left(Z_{ij}\right)}_{mn}$, and the standardized matrix formula is shown in Equation (8).

(8) $$Z_{ij}=\frac{a_{ij}}{a_{1j}}$$

The proportion of the i-th sample under the j-th indicator was calculated as shown in Equation (9), and it was treated as the probability used in the calculation of information entropy.

(9) $$P_{ij}=\frac{Z_{ij}}{\sum_{i=1}^{m}Z_{ij}}$$

The information entropy *e*_j_ of each indicator was calculated, and the information utility value *d*_j_ was also calculated. The calculation formulas are shown in Equations (8) and (9).

(10) $$e_{j}=-\frac{1}{\ln(m)}\sum_{i=1}^{m}P_{ij}\ln(P_{ij})$$

(11) $$d_{j}=1-e_{j}$$

The weight *W*_j_ of the water stability index was calculated as shown in Equation (12).

(12) $$W_{j}=\frac{d_{j}}{\sum_{j=1}^{n}d_{j}}$$

The weights of the key evaluation indicators are presented in Table 5.

According to the entropy method, the weighting relationship of the performance indicators of HAC under water immersion is as follows: tensile strength > bending modulus > bending strength.

### 4.3. Life Prediction

Tensile strength, bending strength, and bending modulus are key indicators of water stability. The *D*-value reflects the comprehensive influence of these individual metrics on water stability performance. The parameter *D* is defined as follows:

(13) $$D_{i}=\sum_{j=1}^{n}Z_{ij}W_{ij}$$

Based on gray system theory, the GM(1,1) model was employed to predict the residual life of HAC under long-term water immersion [45,46,47], with *D*-values represented as original data sequences.

(14) $$X^{\left(0\right)}=\left\{x_{1}^{\left(0\right)},x_{2}^{\left(0\right)},x_{3}^{\left(0\right)},x_{4}^{\left(0\right)},x_{5}^{\left(0\right)}\right\}$$

The first-order accumulated generating operation sequence of *X*^(1)^ is expressed as

(15) $$X^{\left(1\right)}=\left\{x_{1}^{\left(1\right)},x_{2}^{\left(1\right)},x_{3}^{\left(1\right)},x_{4}^{\left(1\right)},x_{5}^{\left(1\right)}\right\}$$

The calculation formula of the moving average is as follows:

(16) $$z^{\left(1\right)}(k)=\frac{x^{\left(1\right)}(k)+x^{\left(1\right)}(k-1)}{2}$$

The first-order whitened differential equation, derived from the GM(1,1) gray differential equation for the accumulated sequence, is established as

(17) $$\frac{dx^{\left(1\right)}}{dt}+ax^{\left(1\right)}=b$$

The initial conditions for the differential equation correspond to the starting index of the modeling sequence, and the initial values are taken as the accumulated values at that index.

The parameter vector E = (a,b)^T^ is defined via the least squares method.

(18) $$E={\left(a,b\right)}^{T}={\left(B^{T}B\right)}^{-1}B^{T}Y$$

The matrix expressions of **B** and **Y** are as follows:

(19) $$B=\left[\begin{array}{cc} -{z_{2}}^{\left(0\right)} & 1\\ -{z_{3}}^{\left(0\right)} & 1\\ -{z_{4}}^{\left(0\right)} & 1\\ -{z_{5}}^{\left(0\right)} & 1 \end{array}\right]\;\;\;\;Y=\left[\begin{array}{c} {x^{\left(0\right)}}_{\left(2\right)}\\ {x^{\left(0\right)}}_{\left(3\right)}\\ {x^{\left(0\right)}}_{\left(4\right)}\\ {x^{\left(0\right)}}_{\left(5\right)} \end{array}\right]$$

By substituting matrices **B** and **Y** into Equation (18), the parameters *a* and *b* can be derived.

The predicted values of the first-order accumulated sequence are expressed as

(20) $${\hat{x}}^{\left(1\right)}(k)=\left\{(\right.x^{\left(0\right)}(1)-\frac{b}{a}\left.)\right\}e^{-ak}+\frac{b}{a},k=1,2,\ldots n$$

By applying the inverse accumulated generating operation to Equation (15), the predicted values of the original sequence are obtained as follows:

(21) $${\hat{x}}^{\left(0\right)}(k+1)=x^{\left(1\right)}(k+1)-x^{\left(1\right)}(k)=(1-e^{a}){\{x}^{\left(0\right)}(1)-\frac{b}{a}\}e^{-ak},k=1,2,\ldots n$$

The relevant parameters of the prediction model are presented in Table 6.

**Figure 15:**
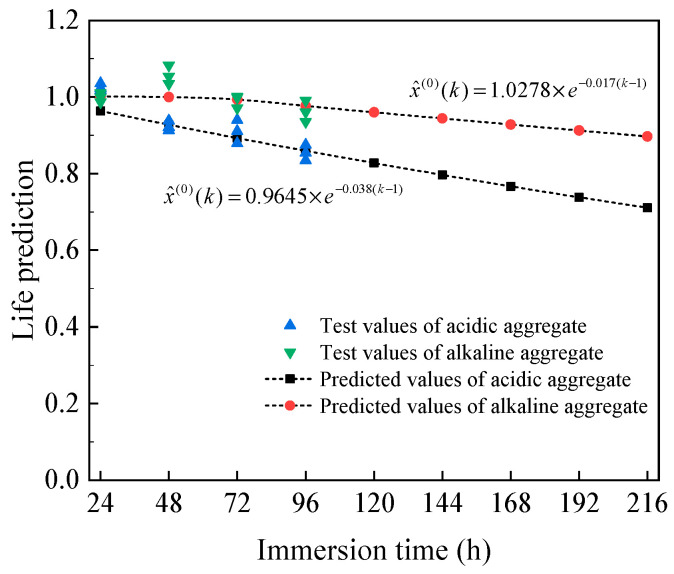
Long-term life prediction of HAC.

Here, *a* is the development coefficient, and *b* is the gray action. “*C*” denotes the mean square error, and “*P*” denotes the error probability. The predicted and test values for the service life of acidic and alkaline aggregates are shown in Figure 15.

Under long-term immersion, the predictive accuracy of the HAC model for acidic aggregates was 98.26%, entering a stable decay phase after 48 h; the predictive accuracy of the HAC model for alkaline aggregates was 97.72%, entering a stable decay phase after 72 h. The results indicate that alkaline aggregate asphalt concrete exhibits excellent water stability in long-term immersion environments and can be used in long-term water environments, while acidic aggregate performs poorly in long-term immersion environments and requires close monitoring [26,44].

### 4.4. Failure Mechanism

Due to the chemical composition differences of aggregates, the water damage degradation of asphalt concrete with acidic and alkaline aggregates shows significant discrepancies. Degradation in short-term water immersion is dominated by interfacial physical peeling. Water rapidly penetrates the asphalt–aggregate interface via capillary action, disrupting the original adhesive layer. Acidic aggregates, rich in hydrophilic silanol groups (Si-OH), readily adsorb water molecules to form a water film, directly inducing asphalt film stripping. Alkaline aggregates, containing Ca^2+^ and other ions, initially show higher adhesion by forming ionic bonds with acidic components in asphalt. Silane coupling agents prove essential for acidic aggregate systems—their hydrophilic termini establish hydrogen bonding with aggregate surface hydroxyls, while hydrophobic ends chemically integrate with asphalt, forming molecular bridges that enhance interfacial anchorage. Although alkaline aggregates possess superior inherent adhesion, exposure to 60 °C hot water induces Ca^2+^ leaching from aggregate surfaces. This generates colloidal deposits that may temporarily enhance strength via “ionic reinforcement effects,” yet concurrent hydration progressively disrupts physical adsorption at the interface, causing gradual adhesive deterioration.

Long-term immersion degradation involves chemical bond fracture, asphalt aging, and crack propagation. In acidic aggregate systems, covalent bonds (Si-O-Si) formed through dehydration condensation between hydrolyzed silane alkoxy groups and aggregate hydroxyls experience partial hydrolysis during extended water exposure. Alkaline aggregate systems demonstrate unique attenuation plateau behavior due to their physical adsorption-dominated interfaces. As immersion duration increases, water infiltration rates diminish while asphalt oxidation crosslinking stabilizes, progressively reducing deterioration rates—a phenomenon attributable to water-resistant barriers formed by carbonate minerals on alkaline aggregate surfaces. Consequently, alkaline aggregate asphalt concrete exhibits superior long-term hydrostability as opposed to its acidic counterpart, despite initial vulnerability.

## 5. Conclusions

This paper conducted compressive, tensile, and bending tests on HAC under different water immersion times. A predictive model was established to predict the remaining life of HAC in a long-term immersion environment. The main conclusions of this study are as follows:

(1) Within a short immersion period, the mechanical properties of asphalt concrete do not exhibit obvious patterns. As the immersion time increases, the mass loss rate gradually increases, the peak compressive stress increases, while the peak tensile and bending stresses decrease. The modulus of deformation generally shows a decreasing trend, while the peak strain varies depending on the acidity or alkalinity of the aggregate.
(2) The correlation between the porosity of HAC and the evaluation indicators of water stability is as follows: tensile strength, bending modulus, and bending strength, with a correlation coefficient greater than 0.8 for all. Tensile strength, bending strength, and bending modulus are used as comprehensive indicators for evaluating water stability.
(3) The GM(1,1) model was used to predict the service life of asphalt concrete long-term water immersion. The predicted values were found to be in good agreement with the actual values, thereby validating the model’s effectiveness. After 192 h of water immersion, the *D*-value of alkaline asphalt concrete degraded to 91.25%, while the *D*-value of acidic aggregate degraded to 73.85%.

## Figures and Tables

**Table 1:** Performance indicators of 70# asphalt.

Penetration (mm)	Penetration Index	Ductility (cm)	Softening Point (°C)	After Thin Film Oven	
				Mass Change	Penetration Ratio
6.2	0.78	21.7	51.1	−0.19%	74.54%

*Note*: The ductility test was conducted at 5 °C with a rate of 50 mm/min.

**Table 2:** Chemical composition of aggregates.

Chemical Composition	Granite Aggregates	Limestone Aggregates
ω(CaO)/10^−2^	0.87	94.72
ω(SiO_2_)/10^−2^	76.10	1.27

**Table 3:** The particle size distribution of the aggregate of HAC.

Mesh Size (mm)	Coarse Aggregate (19~4.75)					Fine Aggregate (2.36~0.15)					Filler
	19	16	13.2	9.5	4.75	2.36	1.18	0.6	0.3	0.15	<0.075
Pass rate (%)	100	93.4	86.6	76.1	57.9	44.1	33.7	26.0	20.0	15.4	12

**Table 4:** Porosity of HAC with acidic and alkaline aggregates.

Aggregates Type	Acidic Aggregates				Alkaline Aggregates			
Immersion time (h)	24	48	72	96	24	48	72	96
Porosity (%)	0.9	0.95	0.99	1.12	0.85	0.97	1.08	1.2

**Table 5:** The weights of the key evaluation indicators.

Weight	Tensile Strength	Bending Strength	Bending Modulus
Acidic aggregate	0.4583	0.1667	0.3750
Alkaline aggregate	0.4232	0.1538	0.4230

**Table 6:** The parameters of the GM(1,1) model.

Model Accuracy	*a*	*b*	*C*	*P*	Model Accuracy
Acidic aggregate	0.038	0.985	0.0087	1	First-level
Alkaline aggregate	0.017	1.036	0.0022	1	First-level

## Data Availability

The original contributions presented in this study are included in the article. Further inquiries can be directed to the corresponding author.
